# Michigan State Board of Health

**Published:** 1883-06

**Authors:** 


### ARTICLE VI.

MICHIGAN STATE BOARD OF HEALTH. (Reported for the CHICAGO MEDICAL JOURNAL AND EXAMINER.)

The Michigan State Board of Health met at its office in Lansing, Mich., on April 11, 1883, the following members being present: Hon. John Avery, M.D., of Greenville; Arthur Hazlewood, M.D., of Grand Rapids; Hon. C. V. Tyler, M.D., of Bay City; Henry B. Baker, M.D., Secretary. Dr. Avery was made President *pro tem.* The minutes of the last two meetings of the Board were read and approved. The secretary read a quarterly report of work in the office of the Board, showing the amount of proof read; meteorological and other reports received; compilation of materal effected; bulletins, documents, circulars, blanks and reports distributed; correspondence performed, etc.; being a brief summary of the work performed in the office of the Board. The secretary also presented a *résumé* of the work performed by other State Boards of Health, and a review of sanitary legislation in other States. He stated that in Michigan the House of Representatives had passed a bill, which was likely to become a law, practically repealing that section of the act establishing the State Board of Health, which provides that the secretary of the State Board of Health shall be the superintendent of vital statistics. If the bill becomes a law, the vital statistics will hereafter be entirely under the control of the Secretary of State. The secretary referred to the alarming presence of small-pox in Nashville and New Orleans, and suggested that Michigan was in danger, because of the approaching time when Southern people flock to the numerous summer resorts in Northern Michigan. The secretary was instructed to correspond with the National and other boards of health, and to do all that can be done to prevent the introduction of small-pox, by quarantine and inter-State inspection. The secretary was authorized to send to health officers in the vicinity of health and summer resorts information of the existence of small-pox in the places whence some of their visitors come; also to send the same notice to health officers of cities especially exposed to introduction of the disease.

The secretary presented an account of sickness caused by eating salted pork. The sickness was attended by burning in the stomach and abdominal tenderness. Some of the meat was fed to four cats. The symptoms in the cats were expansion of the pupils, vomiting, great thirst, and tenderness of the muscles. Diarrhœa was not present. Three of the cats died, the fourth one being barely able to walk after one month. They were attacked twelve hours after eating the meat. A partial microscopical examination of some of the meat by Prof. T. J. Burrill, of Champaign, Ill., disclosed nothing within the meat to have caused the illness, but on the surface of the "lean" portions there was found a micrococcus enormously numerous, as well as some fungous developments of a mould-like kind, sparsely present. The micrococcus was of a new variety, entirely distinct from that of "hog cholera," which latter was not detected in the specimen. It is not known whether the organism was on the pork when it was used for food, and it has not yet been determined whether it is now alive. Culture experiments will be instituted to determine that point. It is quite devoid of motion, and has a less dense or firm appearance than most of its congeners. It takes the ordinary aniline violet stain. Usually two were connected in a figure 8 form; rarely more. The secretary presented statements from Mr. Love, clerk of the board of health of Grand Rapids, showing successful prosecution for selling diseased meat in several cases.

In the afternoon session, Prof. R. C. Kedzie, formerly a member of the State Board of Health, made a report of his attendance at the meeting of the Sanitary Council of the Mississippi Valley, at Jackson, Miss., April 3, 1883, at which he represented the Board. He reported that the meeting was unanimous in urging the continuance of the National Board of Health, and the system of river and railroad inspections, to insure the prevention of the spread of epidemic diseases, without the obstruction of travel and commercial relations between the States, and of such surveillance of the port of New Orleans as will give to the several State and important municipal health authorities represented in the Council, prompt and reliable information of the occurrence and progress of an epidemic disease in that city. Plans for such work were discussed and adopted, and it was understood that in case of the failure of the national government to carry it out after June 2, beyond which time the National Board of Health will be unable to sustain it, unless President Arthur places the $100,000 appropriation at its disposal, it will become important and necessary for the several States endangered to supply, in some way, the money to carry on the work. Delegates from Mississippi, Arkansas, and other States, pledged certain amounts, in case it proves to be necessary. (Michigan has never made an appropriation available for use in preventing the *introduction* of contagious diseases.) Dr. Kedzie received a vote of thanks for the able manner in which he represented the Board at the meeting.

This being the annual meeting of the Board, the election of a President for the ensuing two years resulted in the election of Hon. John Avery, M.D., of Greenville.

The secretary presented an account of the death of a railroad employè by being caught in a "frog," together with a copy of a bill now before the legislature, providing for a wedge of hard wood, or other substance of equal utility, in all "frogs" of an angle of less than 45 degrees. He described the method devised by a prominent railroad man, of tamping hard coal and cinders in the frog. This method is not dangerous to the traveling public, and the wedge of hard wood, by its liability to be misplaced, might throw a train from the track and cause many deaths.

The secretary was authorized to prepare a blank form for the use of health authorities in Michigan in reporting the occurrence of a disease dangerous to the public health, to the State Board of Health.

Invitations to hold sanitary conventions at Muskegon and Ionia were accepted, the dates to be hereafter decided upon.

The newly elected President announced the following

STANDING COMMITTEES.

1. Epidemic, Endemic and Contagious Diseases.—H. F. Lyster, M.D.

2. Sewerage and Drainage.—H. F. Lyster, M.D.

3. Food, Drinks, and Water-Supply.—V. C Vaughan, M.D.

4. Buildings, including Ventilation, Heating, etc. — Jno. Avery, M.D.

5. Climate, Geology, Topography, etc.—Henry B. Baker, M.D.

6. Disposal of Excreta.—John H. Kellogg, M.D.

7. Poisons, Explosives, etc.—V. C. Vaughan, M.D.

8. Occupations, Recreations and Habits.—J. H. Kellogg, M.D.

9. Relations of Schools to Health —Jno. Avery, M.D.

10. Sanitary Survey.—C. V. Tyler, M.D.

11. The Death-Rate as Influenced by Age. — Henry B. Baker, M D.

12. Legislation.—C. V. Tyler, M.D.

13. Finances of the Board.—Arthur Hazlewood, M.D.

14. Mental Hygiene.—Arthur Hazlewood, M.D.

15. Diseases of Animals.—Henry B. Baker, M.D.

16. Relations of Preventable Sickness to Taxation.—John H. Kellogg, M.D.

These nominations were confirmed by the Board.

Dr. Baker, as committee on diseases of animals, reported an effort being made in the legislature to secure an appropriation for the State Board of Cattle Commissioners. Dr. Tyler reported the presence of a bill in the legislature prohibiting putting sawdust into streams.

Dr. Hazlewood reported that he had frequently seen old oil barrels being collected in Grand Rapids, from which the brand had not been erased, and often, on new invoices of oil, the inspector's brand was so indistinct that the date of inspection could not be deciphered.

The secretary was requested to prepare a memorial to the President of the United States, petitioning that he place the $100,000 appropriation in the hands of the National Board of Health.

The Board performed much routine business, and adjourned to meet at Reed City, Michigan, April 26 and 27, 1883, at which time there will be a sanitary convention at that place under its auspices.

